# A Human Digital-Twin-Based Framework Driving Human Centricity towards Industry 5.0

**DOI:** 10.3390/s23136054

**Published:** 2023-06-30

**Authors:** Gianfranco E. Modoni, Marco Sacco

**Affiliations:** 1 Institute of Intelligent Industrial Systems and Technologies for Advanced Manufacturing, National Research Council, 70124 Bari, Italy; 2 Institute of Intelligent Industrial Systems and Technologies for Advanced Manufacturing, National Research Council, 23900 Lecco, Italy; marco.sacco@stiima.cnr.it

**Keywords:** Industry 5.0, human digital twin, collaborative intelligence, human centricity, Operator 5.0

## Abstract

This work presents a digital-twin-based framework focused on orchestrating human-centered processes toward Industry 5.0. By including workers and their digital replicas in the loop of the digital twin, the proposed framework extends the traditional model of the factory’s digital twin, which instead does not adequately consider the human component. The overall goal of the authors is to provide a reference architecture to manufacturing companies for a digital-twin-based platform that promotes harmonization and orchestration between humans and (physical and virtual) machines through the monitoring, simulation, and optimization of their interactions. In addition, the platform enhances the interactions of the stakeholders with the digital twin, considering that the latter cannot always be fully autonomous, and it can require human intervention. The paper also presents an implemented scenario adhering to the proposed framework’s specifications, which is also validated with a real case study set in a factory plant that produces wooden furniture, thus demonstrating the validity of the overall proposed approach.

## 1. Introduction

While the push of Industry 4.0 is still active to increase the flexibility and efficiency of the production processes, a new paradigm is already emerging on the horizon in the manufacturing scope. But what are the motivations behind a further paradigm shift that goes beyond Industry 4.0 (I4.0)? First, Industry 4.0 is a technology-centered and technology-driven paradigm. Thus, it is more focused on digitalization and AI-driven technologies and less on other relevant principles such as social fairness and sustainability [1]. In addition, although various studies analyzed the links between new digital technologies and human factors [2,3], their interrelations and the sociotechnical impact deriving from these interrelations have been so far insufficiently addressed. To address these criticalities of the Industry 4.0 model, institutions and policymakers are starting to shift their attention toward human-centered design and ethical and responsible innovation in the factories of the future. In this regard, various research studies that venture in these directions are ongoing (or have just been completed) and may all be brought back to the umbrella term of Industry 5.0. The latter aims at complementing the Industry 4.0 paradigm to allow the industry workers to return to the center of all the production processes. The European Commission (EC) conceives the paradigm shift towards Industry 5.0 as based on three core elements: sustainability, resilience, and in particular, human centricity [1]. This new vision of human-centric manufacturing aims to “place human well-being at the centre of manufacturing systems and processes, providing a safe, comfortable, and motivating environment for working, learning and growth” [4], thus overcoming the Industry 4.0 technology-driven model which instead places system optimization at the core of manufacturing.

Despite the push toward the Industry 5.0 topic, its concept and form remain unclear [5]. In particular, the vision of human centricity as designed by the researchers of EC and also of other institutions is still abstract, lacks significant practical cases, and requires an in-depth study of its implementation aspects. In order to move toward a human-centric scenario, the cooperation between machines and human beings needs to be better exploited by combining their diverging strengths while removing their weak points [6].

To create the conditions of this paradigm shift, efforts should be directed toward identifying new strategies to search for comfortable and efficient collaboration mechanisms between humans and machines to allow both to provide mutual situational assistance (in addition to the already existing situational collaboration) [4]. In this new context, workers no longer need to adapt to machine requirements as within the traditional shopfloor, but on the contrary, machines must be conceived so that they are able to recognize users; remember and take into account their capabilities, skills, and preferences; and adapt accordingly [7]. The development of such kinds of machines requires extensive research that needs to consider, study, and also combine various enabling technologies [8]. 

One of these enabling technologies is the digital twin (DT), which is considered a building block of future manufacturing to virtually represent assets, products, and other resources, thus allowing researchers to simulate performance, predict failures, or investigate problems within the factory [9,10]. In this regard, in order to contribute to identifying practical operational lines to implement a human-centric approach in manufacturing, this work presents a human DT model for a collaborative intelligence interaction framework (from here on called Human-CENTRO), which is a DT-based architectural framework that integrates humans, physical entities, and the cyber world of the DT. The overall aim of this work, conducted within the European research project AI REGIO (“Regions and Digital Innovation Hubs Alliance for AI-Driven Digital Transformation of European Manufacturing SMEs”) [11], is to promote the orchestration of worker and machine activities through the monitoring, simulation, and optimization of their interactions. In particular, this framework considers and manages the two interaction patterns that occur between humans and machines according to the collaborative intelligence (CI) model (i.e., “Humans Train Machines” and “Machines Assist Humans”) [12], where the involved machines can be both physical machines (e.g., robots, CNC machines, etc.) and digital components (e.g., AI application, etc.).

Since the traditional model of the factory’s DT does not adequately consider the human component [13], this traditional model is extended within Human-CENTRO to take into account the impact of CI interactions. Moreover, also considering that a DT cannot always be fully autonomous, and it can require human intervention [14], the framework also assesses how DT’s architecture can be complemented and work with humans.

Finally, the paper presents an implemented scenario adhering to the framework’s specifications, which is also validated with a real case study set in a factory plant that produces wooden furniture, thus demonstrating the validity of the overall proposed approach.

The proposed framework is intended to be used as a reference architecture for a fit-for-purpose DT-based platform that takes into account and manages the CI interaction between workers and machines, thus guiding researchers and technicians to prepare strategic future developments and deployments of a human DT toward human centricity in manufacturing.

The remainder of this paper is organized as follows: Section 2 reviews the existing works in the literature about the main topics involved in the current study. Section 3 elicits the framework’s requirements starting from a motivational scenario. Section 4 introduces the architectural frameworks, while Section 5 presents the implementation of the framework into the motivational scenario, thus demonstrating the validity of this approach and the opportunity to adopt the framework. Finally, in the last section, we draw conclusions summarizing the main outcomes and outlining future lines of investigation.

## 2. Related Works

### 2.1. Frameworks, Conceptual Models, and Solutions for Human-Centered Factories

The realization of the vision of Industry 5.0 requires research in different fields and also the combination of several technologies. In this regard, a survey on challenges and supporting technologies for Industry 5.0 devoted to both industrial and academic communities is provided in [15]. Similar topics are covered in [16], where the authors, through a critical literature review, aim to identify which fundamental components and technologies enable the transition from Industry 4.0 to Industry 5.0. In [17], the authors have also exploited text-mining techniques and tools to analyze the published literature and classify the concept of Industry 5.0, with the overall goal to understand the evolution of Industry 5.0 and its enabling technologies.

Regarding the frameworks already available in the literature, a generic system architecture for realizing Industry 5.0 based on three dimensions (technical, reality, and application) is proposed by Leng et al. in [18]. However, it represents only a basic theoretical architecture that, according to the intentions of the authors, aims to stimulate the discussion about architecture for Industry 5.0.

Other recent studies mainly focus on one specific aspect of the three pillars of Industry 5.0. In particular, Longo et al. address the specific social sustainability requirement, by reporting the growing concern in the modern industry about the ethical issues that arise with the adoption of new technologies toward the realization of the factory of the future [19]. In this regard, the authors propose a framework that helps to take into account these issues during the use of the new technologies. Instead, Romero et al. aim to provide the resilience capability to both workers (to overcome their human weakness) and to all human–machine systems (to optimize their cooperation) [20]. Specifically, workers’ resilience concern their biological, physical, cognitive, and psychological health and safety in the workplace, while system resilience refers to the ability of adjustable autonomy and the shared control of human and machine systems to ensure high performance in their cooperation.

To complete the new Industry 5.0 vision, as far as human centricity is concerned, recent works that take into account some aspects of this topic mainly focus on human factors and ergonomics [21,22], Operator 4.0 [23], Operator 5.0 [24], and human–robot collaboration [25]. However, all these works only partially reflect a human-centric vision since they continue to be still enclosed in a technology-driven context. Indeed, in all these works, humans and machines are expected to cooperate in shared workplaces through synchronized activities, and, in such scenarios, the system’s performance and optimization are still posed at the center of manufacturing as provided by the Industry 4.0 paradigm [4].

A work that provides a perspective on human-centric assembly is reported in [26]. This perspective leverages four enhanced human abilities to energize, advise, support, and empower a human operator physically and intellectually. The provided perspective is very interesting, but it is futuristic also in consideration of some of the technologies on which it is based (e.g., brain robotics). Various recent works review the challenges that need to be addressed to realize the vision of human centricity in practice. In particular, a systematic review that analyzes the main strategies, challenges, and enabling technologies for the implementation of human centricity in future industrial environments is conducted in [27]. Another similar review is conducted in [28], an article that takes into account and analyzes human-centric oriented solutions (and corresponding technologies) applied in fields like healthcare, supply chain, and production in manufacturing. In addition, the challenges in the implementation of human centricity are detailed in [29], where these challenges are introduced through the analysis of a case study where workers and robots collaborate synergically. Through this case study, the main key features of a human-centric vision are also outlined. Moreover, a recent article presents a roadmap that traces the next steps for the development of the human–machine relationship oriented toward Industry 5.0, and this roadmap is based on both the author’s experience and a literature review [30]. However, all these mentioned studies limit the analysis to the concept of human centricity at a fairly high level, but they do not implement solutions for the examined challenges. The vision of human centricity designed by the EC researchers in [1] is abstract and lacks significant practical cases. Under these conditions, among the various still open challenges emerges the need to identify a conceptual model that guides the implementation of a human-centric approach within the factory, which is instead the overall goal of the herein-presented work.

Another work that goes in the direction of a human-centric scenario is reported in [4]. This work proposes a technological reference model that abstracts the architectural and functional components of a human-centric manufacturing system for realizing empathic machines that, by sensing emotions, needs, and preferences, can provide situational assistance to humans. The three provided components of the model are human–machine understanding, human–machine communication, and collaborative intelligence. Since this reference model remains at an abstract level and far from practical application in a real case, it will be interesting to apply it to some probative real-use cases for Industry 5.0 implementation. In this regard, some interesting case studies available in the literature and focused on Industry 5.0 can be exploited to evaluate its correctness. These case studies are mainly set in the fields of brain robotics [26] and collaborative robotics [29]. Among other things, the objective of testing a conceptual model oriented toward Industry 5.0 within real scenarios is pursued in the work presented in this manuscript. In this work, the proposed framework exploits as starting point to implement the human-centric vision; one of the three pillars of the approach presented in [4], i.e., the CI; and, specifically, the CI interactions as conceived in [12].

This CI model envisages two collaborative interaction channels. In one direction, i.e., “Humans Assist Machines”, the goal is to train machines to perform specific tasks, explain the results of those tasks, and sustain the responsible use of machines. In the second direction, i.e., “Machines Assist Humans”, the goal is to amplify human cognitive strengths and physical capabilities, interact with customers and employees, and embody human skills to extend their physical capabilities. Each of the two interaction channels of the CI can be of interest in the scope of a specific scenario depending on the case study’s requirements. In this regard, the proposed framework in this study aims to support the analysis of these requirements by considering the involved interactions and also offers a technological architecture to support them.

It should be noted that the enabling technologies of human centricity are a set of complex systems that combine technologies with sensors [31]. One of these technologies is the DT model, which can be exploited to optimize production, test products, and processes and detect possible harmful effects. Its relevance for Industry 5.0 is also confirmed by other recent studies (e.g., [18,32]). In this regard, the overall potential of the DT is more thoroughly analyzed in the following section.

### 2.2. The Worker in the Loop of DT

#### 2.2.1. Introduction to DT

**Figure 1:**
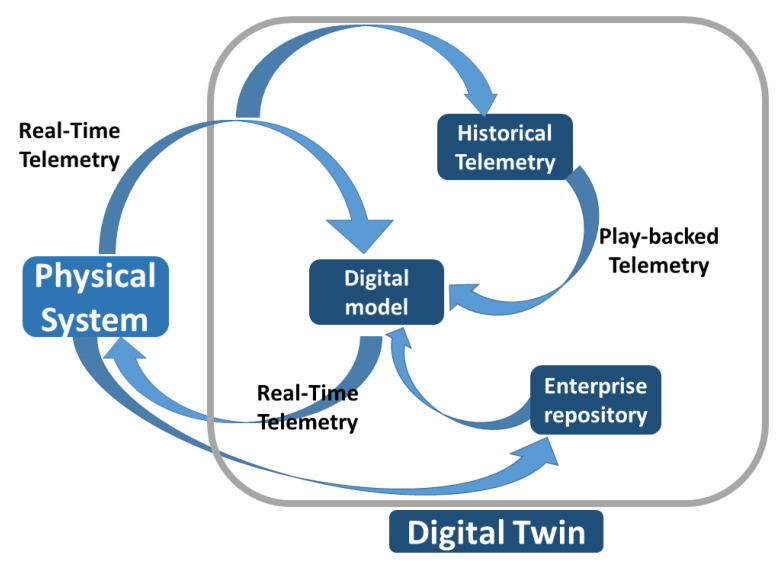
Data pipelines within the traditional conceptual model of DT.

The DT is an integrated multi-physics, multi-scale, probabilistic simulation of an as-built system that uses the best available physical models, sensor updates, fleet history, etc., to mirror the life of its corresponding twin [33]. Under these conditions, it provides a representation of the characteristics and behavior of its physical counterpart according to a specific level of fidelity. The general idea is that a DT-based approach can augment physical assets, by extending them with a kind of shadow in the digital space. It should be underlined that the DT becomes a real replica of its physical counterpart when it is fully synchronized with it through a circular process across the physical and virtual worlds (Figure 1). Indeed, in this case, it is possible to mirror the real operating conditions, by allowing for the simulation of real-time behavior, the prediction of performance indicators, the anticipation of failures, and the planning of prompt interventions to mitigate damage or degradation.

The potential of DT in manufacturing has been analyzed in several scientific articles and publications [34,35]. In addition, various methodological approaches have been described, and various solutions have been implemented by leading technology providers such as Siemens, Beckoff, and Microsoft DT.

#### 2.2.2. The Traditional Conceptual Model of Digital Twin

Figure 1 illustrates the traditional conceptual model as typically represented in the literature [36]. It puts in evidence the continuous synchronization between a real physical asset and its digital counterpart, which is realized by means of two streams of data. The first one (from left to right) represents the real-time-monitored data flow and includes all the physical variables sensed at the physical level by ubiquitous sensors and transmitted to the digital space. Conversely, the second stream (from right to left) involves actions to be performed in real time or near real time at the shop floor level, representing the feedback returned in the form of low-frequency waves from the digital space to the real factory, e.g., corrective actions and planning decisions that can be the result of the execution of control algorithms.

Under these conditions, the traditional conceptual model of DT comprises four major components: the digital model, the enterprise repository, the factory telemetry, and the digital thread (Figure 1) [36]. The first component is an abstract characterization of the asset with all its parts and logical relations existing between parts, and it also contains a description of its behavior. The second component is the enterprise repository, a database that contains all the information concerning the management of the organization’s resources. The third component is the factory telemetry, which allows for the continuous bidirectional synchronization between the physical artifact and its digital counterpart, i.e., a constant mirroring of the two sides thanks to two opposite streams of data in motion.

Finally, the digital thread or historical factory telemetry, the fourth component, is the temporal (or historical) extension of the twin, which keeps track of the evolution of the physical twin by accumulating and storing (at rest) the data acquired at the real level. It should be noted that the historical factory telemetry is historicized on a specific database and then used as an input to the model (through appropriate playback of data in motion) to perform predictions against which to compare the behavior of the real system. Additionally, historical data can be used to make comparisons to the current telemetry.

A significant shortcoming that emerges from a study of the current state of the art of DT is that the focus of the DT solutions available in the literature is almost entirely on physical assets and not on human operators [37]. Moreover, it should be noted that this traditional model of DT does not adequately consider the presence of workers, which in many cases is relevant within the DT loop [13]. In fact, one of the initial motivations behind the creation of a DT for a physical asset was the need for a system capable of monitoring, processing, and making some decisions of corrective actions to be applied to the real asset, automatically and without human intervention (Figure 1). Under these conditions, the traditional model of DT, which neglects the human component, cannot be compliant with the concept of Industry 5.0, which instead foresees the centrality of the worker. In order to contribute to bridging this gap, the traditional model of DT is extended within Human-CENTRO to take into account the impact of CI interactions. Moreover, besides the evaluation of the interactions of workers with physical machines, the proposed framework can also contribute to assessing how DT’s architecture can be complemented and work with humans, by enhancing the interactions of stakeholders with the DT. In fact, the latter is not always fully autonomous and may require human intervention [14], particularly in scenarios where they are used to test new features and modifications of physical assets, or when they are needed to provide answers such as treatments and diagnosis [38].

## 3. A Motivational Scenario: The Augmented Operator 5.0

To better understand the objectives and key enabling factors of the framework, it is essential to focus on the analysis of practical scenarios that can help understand and elicit the requirements of the Industry 5.0 framework. In this regard, a production line set in an Italian factory plant that produces wooden furniture offers a valid scenario to highlight the motivations behind this study. In this factory, the production layout includes several working stations that can perform different operations (e.g., milling, drilling, etc.), and each worker is usually trained to accomplish specific tasks (e.g., assembly of specific parts, etc.). As workers are not very interchangeable within the assembly line, the company cannot apply a complete job rotation. Under these conditions, it has great difficulty in balancing the workload, especially when it has to deal with peaks of requests for specific products or in the case of unavailability of some workers.

Moreover, the activities of workers are hindered by the use of hard-copy manuals for work instructions in assembly operations that force them to check, at the beginning of each new assembly, the instruction sheets of the product. These hard-copy manuals entail extra effort for workers who also have to deal with changes in work instructions due to any revision of the product. This approach can lead to time waste, which mainly depends on the experience of the worker and the frequency of the production of different furniture models.

This difficult scenario is worsened by the fact that, in the same workplace, there may be various potentially dangerous situations for workers, deriving, for example, from the crossing of workers with moving machines (AGV or loader/unloader robots) or from their overexposure to the heat, UV radiation, or noise generated from specific working machines. Therefore, workers must pay attention to the various risks to which they are exposed during their working activities.

The above-described scenario advocates the need to continuously align workers’ skills and competencies, as well as their knowledge concerning safety procedures, to the changing requirements of the company. This need can be supported by means of an on-the-job training approach that is applied within the real workplace while manufacturing processes are in progress. In this regard, this study investigates the potential of the CI approach and its “Machines Assist Humans” pattern interaction in order to train workers by providing them with just-in-time information targeted to each specific individual depending on their level of knowledge and skill. This information also aims to make machining and transport activities safer for the involved workers while maintaining the effectiveness and efficiency of the processes. In addition, the machines that collaborate with workers must be able to react to mistakes made by workers during task completion by recommending the right intervention to correct their mistakes.

## 4. Human-CENTRO: A DT-Based Framework for Human-Centered and CI Processes

The Human-CENTRO framework aims to support human-centered scenarios set in contexts where physical machines and AI-driven systems interact with humans according to the CI paradigm. One of the main goals of this framework consists of providing a valid solution to support the orchestration, optimization, and simulation of human-centered processes oriented toward Industry 5.0.

Since the DT is becoming a consolidated technology to simulate the physical industrial asset performance, allowing for the prediction of failures or the investigation of problems, this work aims to investigate the potential of a DT-based solution for optimizing human-centered processes. Specifically, the DT represents a virtual and faithful mirror of the physical process that allows for the monitoring of process parameters, their comparison with any analytic models, and the provision of real-time specific parameter variations to keep the process always in optimal conditions. Under these conditions, the DT can provide the backbone for the real-time monitoring of the current human-centered processes to enhance their efficiency, update decisions about resource management, predict failures, and assist in the implementation of optimization strategies. Thus, leveraging a human DT-based solution, Human-CENTRO can contribute to refining the collaborative interactions between humans and machines by promoting the harmonization and orchestration of these interactions. In particular, this framework can enable an AI-based digital workplace that is both immersive and pervasive, providing augmented workers and managers with on-demand capabilities tailored to their needs and preferences.

### 4.1. Collaborative Intelligence Interactions within the DT Model

**Figure 2:**
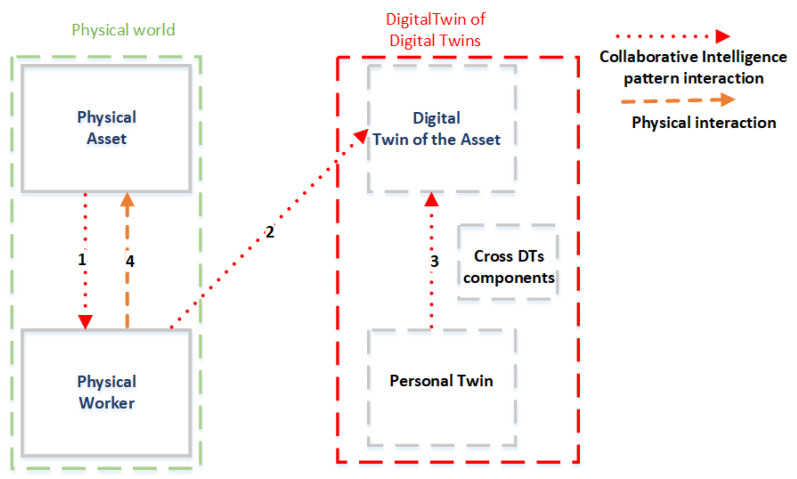
CI interactions within the new DT model.

**Figure 3:**
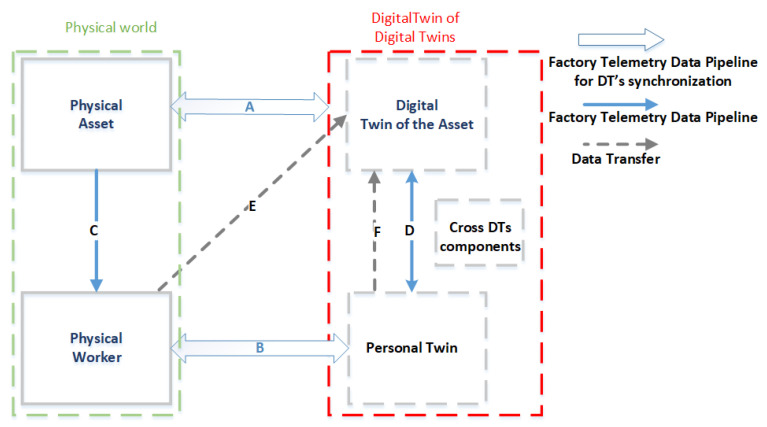
The evolution of the DT model: data pipelines for supporting CI interactions.

In this section, the impact of the CI concept on the traditional DT model is discussed. In this regard, the study investigates the new blocks that must be introduced within the DT conceptual model and the CI interactions that involve these blocks. These blocks and interactions are illustrated in Figure 2. In particular, the figure introduces, next to the blocks of physical assets and their DTs, the blocks corresponding to physical workers and their corresponding personal twins (PTs). PTs are in turn combined and integrated with all the other DTs in a federation of DTs, creating the conditions to realize a DT of DTs (red hatched rectangle in Figure 2 and Figure 3).

These new blocks can in turn be complex macro-blocks that can include various components. In particular, the PT can comprise two macro-components: (1) the model representing the information of the physical worker; (2) the behavioral cognitive model. The DT of DTs also includes some further components (named cross-DT components), which are transversal to all the DTs within an organization in order to offer them specific functionalities. One example of a cross-DT component is a security service that provides various functionalities that guarantees the security of all the DTs within an organization.

In addition, the proposed model introduces interactions among different blocks and derives from the two interaction patterns of the CI model. These interactions are represented in Figure 2 as red dotted arrows, and the definition of each of these arrows is also preliminarily summarized in Table 1.

Specifically, the interaction pattern “Machines Assist Humans” is generated through a link between the physical system and the physical worker (arrow 1). Examples of this interaction can be realized through an exoskeleton or AR viewer. This interaction pattern can be expressed as the following function:

MachineProvidesPhysicalSupportToHuman (PWj, PTj)(1)

which takes the physical worker who must be assisted and their PT as input parameters in order to take their needs into account.

Conversely, the interaction pattern “Humans Assist Machines” is generated through a link between the physical worker and the DT of the machine (arrow 2). This interplay can in particular take place through digital interaction (e.g., data entry, etc.) or through the voice interaction of the worker and the machine. Through this interaction, stakeholders can transfer expert domain knowledge to machines in the form of data models to train the model of the DTA. According to the above description, this interaction can be expressed as the following function:

InjectModelsIntoDT (DTToUpdate, {model1, …, modeln})(2)

which takes as input parameters the DT to be updated and a list of models that must be used to update the DT.

Finally, the new proposed DT model also considers the hybrid cognitive DT, which can add cognitive elements to the control systems [39]. Indeed, the cognitive DT can be realized through a CI interaction since a subset of the functionalities of cognitive models of the PT is transferred to the DT of the system (shown with arrow 3), and here, part of the PT is revived in one of the submodels of the DT of the system.

This interaction can be expressed as the following function:

TransferCognitiveModel (DTA, {PT1, …, PTk}) (3)

where the input parameters are the DT to be updated and a list of models that must be exploited to create the cognitive DT of the asset.

To complete the analysis, for the convenience of the reader, some examples of enabling technologies for the two pattern interactions are summarized in Table 2. Each technology mentioned in the table is differentiated by the type of machine (physical or digital) and is also coupled with the arrow that characterizes the specific interaction defined in Table 1.

### 4.2. Data Pipelines to Support the CI Interactions within the DT Model

Owing to the CI interactions described in the previous section, humans and machines work and interact collaboratively within the workplace. Since these interactions are also supported by new streams of factory telemetry data, in this section, we analyze the data pipelines through which information flow occurs between two different components of the proposed model. In Figure 3, which represents the evolution of the DT model, these pipelines are represented as solid blue uni- and bidirectional arrows, which are preliminarily summarized in Table 3.

In particular, the bidirectional arrow A corresponds to the factory telemetry component of the traditional model of DT as described in Section 2.2.2. Thus, arrow A represents the process of synchronization between the physical asset and its DT (DTA), while the bidirectional arrow B represents a new telemetry stream that enables synchronization between new blocks of the physical worker and its digital personal twin.

In addition, the visual interaction of the worker, which allows for the demonstration of information from the DTA to the worker (e.g., through an AR viewer, a monitor, or another physical device), implies a connection between the physical system and the physical worker (the unidirectional arrow C).

Moreover, to allow a machine to adapt to the needs of a specific person with whom the machine is collaborating, the DT of the machine must access the information of the person included in the PT. In this regard, the bidirectional arrow D represents the interplay between the DTA and the PT and vice versa. For example, this link is essential if the DTA contains model-based analytics, which also needs the data of the person for its processing and elaboration.

In addition, exploiting the pipeline represented by arrow E, the pattern “Humans Assist Machines”, described by arrow 3 in Figure 2, is implemented, which allows for the training of the learning model of the DTA or the transfer to the machine’s expert domain knowledge under the form of models.

Finally, arrow F represents the transfer of the cognitive models of the PT to the DT of the system.

In summary, the changes applied to the traditional model of DT to support human centricity can be highlighted by comparing the diagrams in Figure 1 and Figure 3. The newly introduced blocks (corresponding to physical workers and their corresponding personal twins) and the newly introduced arrows (B, C, D, E, and F) are the distinctive elements of the herein-proposed human DT model when compared to the traditional DT model.

To conclude this section, it should be noted that one of the aims of this study is the identification of a valid software infrastructure capable to support automatic data pipelines. The affected links are in particular the arrows labeled as A, B, C, and D in Figure 3.

### 4.3. Considerations in Human-CENTRO

The following additional considerations on Human-CENTRO can be reported:

- Arrow 4 in Figure 2 represents a physical interaction that is not part of the set of CI interactions. It links the physical worker with the physical system and can occur when the worker acts on a haptic device, a mechanical device, or even a keyboard.
- In the proposed model, there is no link between the DTA and the physical worker. Indeed, the information included within the DTA must be conveyed to the physical worker through a physical device (such as an AR viewer), by exploiting the data pipeline represented by arrow C in Figure 3.
- Since the PT comprises two macro-components (i.e., the model representing the information of the physical worker and the behavioral/cognitive model), it could be interesting to investigate how the two macro-components are linked and/or how they are integrated with the DTA. Indeed, workers interact with each other and with machines, also through DTs.
- Which of the represented flows (arrows) are affected by an orchestrator component, which plays the role of balancing between the human component and machine? The affected links are 2 and 3 in Figure 2, as well as A, B, and D in Figure 3.
- As underlined in Figure 3, the data do not flow only in a circular process as in the traditional DT model. Indeed, since the enabling technologies of CI could even be brainwaves, brain impulses are transmitted from the physical worker to their PT or even directly to the DTA.
- Since the use of explainable AI [45], i.e., a set of tools and frameworks that help to understand and interpret the predictions made by machine learning models, is becoming increasingly common, what is their impact on this proposed model, and what are the pipelines that support these explanations? Explainable AI extends the interaction pattern of “Machines Assist Humans”, and two options can be considered in the proposed model to manage the explanations sent from the DTA to the worker: (1) the same pipelines used for other data (arrows A and C in Figure 3) are exploited; (2) the explanations are sent using ad hoc pipelines that are used only for this goal.

## 5. The Pilot Study

This section introduces a lightweight prototype of a solution that has its root in the Human-CENTRO’s architecture. Specifically, we investigated the Human-CENTRO’s potential within the motivational scenario reported in Section 3 to assess the framework’s capability to support real scenarios.

To this end, a technological solution was implemented that would be able to recognize the profile of each worker and remember their capabilities, skills, and preferences with the final goal of guaranteeing them personalized and always up-to-date information. This information can be visualized within real scenarios to augment the operator’s perspective, allowing them to receive a set of information depending on their background and guiding the specific task that is being performed. The proposed solution must also be able to recommend the right intervention in order to remedy possible errors. In addition, it provides the information needed for the worker to avoid collisions and detect the restriction enforcement rules of potential danger zones (e.g., workers close to a dangerous working machine).

**Figure 4:**
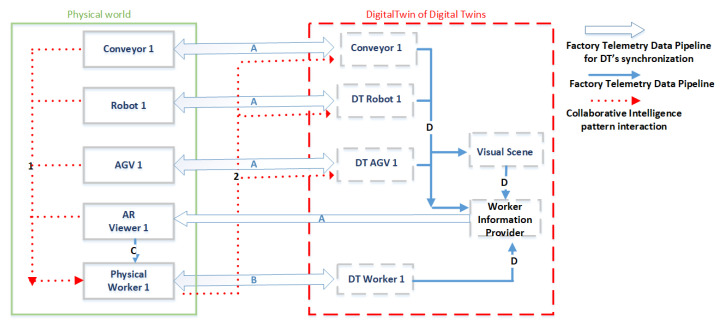
The application of the conceptual model of the motivational scenario described in Section 3.

The solution comprises the following major components (Figure 4):

1. A distributed set of smart devices monitoring the position and status of different resources on the shop floor;
2. A set of PTs (one for each worker) representing the worker and in particular including their biographic info, skills, and competencies;
3. A set of DTs, each corresponding to a physical asset present on the shopfloor. Indeed, each resource has a corresponding synchronized (physics or data-based) model within its DT, which reproduces its behavior;
4. A worker information provider (WIP), which is a rule-based data-driven component that selects and manages the personalized information to be visualized for workers using an AR application in order to guide their tasks;
5. Visual scene analysis, which is a specific software component to manage safety zones by enforcing geofencing boundaries and danger zone restrictions. In this regard, it includes functionalities such as the management of unauthorized access. The proximity to restricted areas, danger zones, or potential hazards (e.g., heat, UV radiation, and noise overexposure) are shown to the workers using an AR application;
6. A mobile application based on AR provides the workers, through the WIP component, with a set of dynamic personalized signs and labels within the real workplace (also in the operating machines) in order to guide their work.

To start a task, the worker uses the AR application to be authenticated in the system, which verifies if they are fit (in terms of skills and capabilities) to perform a specific task. Afterward, the AR application guides the worker step by step to accomplish their task by presenting targeted information. In particular, the application offers different levels of support for three different groups of assemblers: workers with limited experience (high support), workers with medium experience (medium support), and the third involving expert workers (low support). Any updates in the manufacturing process (e.g., a new product) necessitate a refresher course for the worker, which is ensured on the job using the proposed solution. In addition, in order to give the worker a clear idea of how to continue the work in progress, the AR application can visualize a preview of the 3D drawing of the finished product by superimposing the mobile device on the part of the product that has been built so far.

### 5.1. Application of Human-CENTRO within the Motivational Scenario

In order to better analyze the CI interactions for the solution proposed within this motivational scenario, in this section, the framework reported in Section 4 is applied, and the results of this application are presented.

For the sake of simplicity, although many resources can be involved in the real scenario, a representant for each category (a robot, an AGV, a conveyor belt, etc.), as well as a generic worker, is presented in Figure 4. Each of the machines is linked with the worker, shown as arrow 1, to represent the idea of machines supporting humans. In addition, each resource is synchronized, through a specific connection, with its corresponding DT, which in turn includes one or more specific models.

Arrow 2 implements the channel “Humans Assist Machines”, which is exploited to transfer knowledge from humans to machines, with the former specifying the models included in the DT of the latter.

In addition, as provided in Human-CENTRO, the WIP must be positioned in the DT ensemble of DTs, since it is transversal to the different DTs. The WIP receives data from the various DTs (e.g., the positions of the asset, etc.), shown as arrow D, while its output is visualized within the AR viewer, shown as arrow A, to be then visualized for the worker, shown as arrow C (visual interaction).

### 5.2. Implementation and Technology Adoption

**Figure 5:**
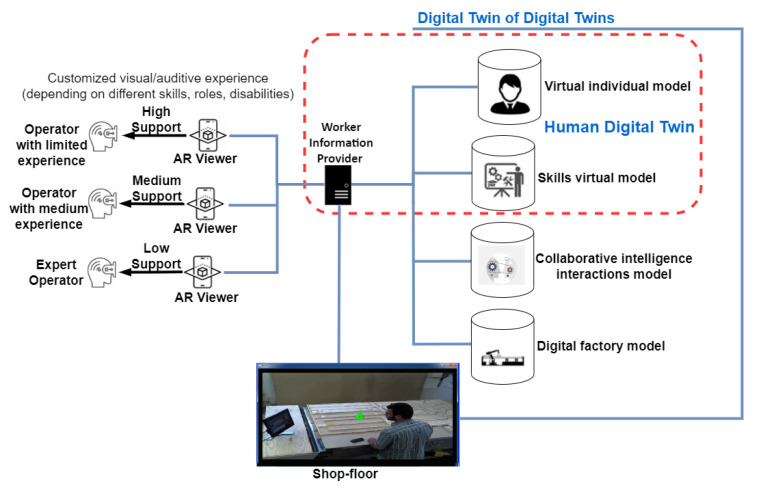
The use of four data models to customize the information visualized for assembly operators.

The backbone of the proposed solution is the meta-models underpinning the DT of DTs. There are four main meta-models, represented using a semantic formalism (i.e., OWL) [46] (Figure 5): (1) the digital factory model (DFM); (2) the virtual individual model (VIM); (3) the skill virtual model (SVM); and (4) the collaborative intelligence interactions model (CIIM). In particular, the DFM abstracts the manufacturing system by representing production processes, products, components, and materials. In this regard, it takes into account and borrows concepts and relations from the virtual factory data model introduced in [47]. The VIM provides a description of the worker profile within the factory, which includes biographic info, capabilities, work aspirations and attitudes, training activities, and courses the worker has already taken part in. It also contains eventual worker impairments, and this information can be used to automatically compensate for these impairments (for example, in case of visual deficit, by adjusting the lighting of the work surface). It should be noted that the VIM is also one of the two meta-models that are behind the human digital twin (HDT) model. The other is the SVM, which provides a representation of the skills the worker needs in order to perform every single task of the production (through a link with the VIM and a link with the DFM). It also includes competencies and operator capabilities by taking into account the skill model defined in the report produced by the European Commission [48]. Finally, the fourth meta-model is the CIIM, which abstracts the CI interactions between humans and machines and is mainly based on the model of the Industry 5.0 data space [49].

**Figure 6:**
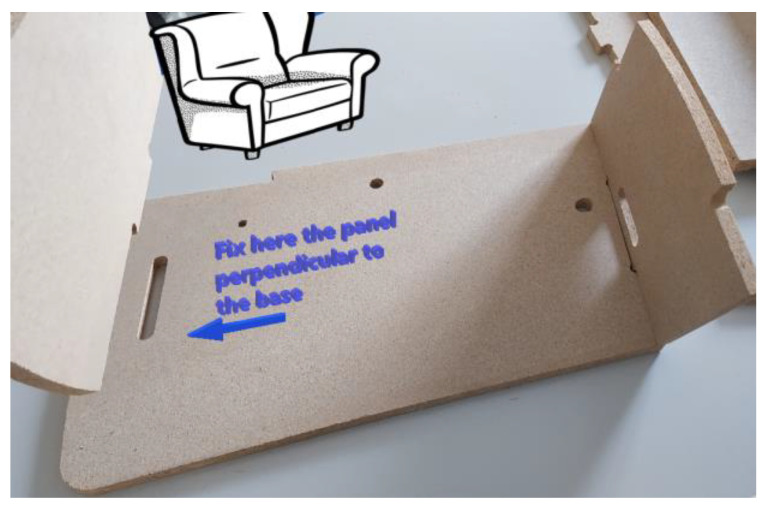
Example of information visualized using the AR application.

The instances of the above-mentioned four meta-models can be in turn updated thanks to the publish/subscribe pattern provided by the adopted middleware, Apache Kafka [50], which thus enables the synchronization of the DT of DTs. Using Apache Kafka, indeed, any client can either publish information (in the form of telemetry) or subscribe to receive specific information by specifying their preferences. In particular, the operating machines publish information about their status, while the distributed position sensors publish information concerning the position of the different monitored resources and also of the workers (in this case, the generated telemetry is part of the HDT of the corresponding worker). The published information can then be consumed by the WIP (also included in the HDT) (Figure 5) with the goal to customize the information to be visualized for the workers. They can see this information with an AR application (Figure 6), which is in turn developed with the Vuforia engine for the Android platform [51] and imported within the Unity platform [52]. Moreover, using visual scene analysis, information concerning the assets’ positions is exploited in order to be visualized within a graphical environment of the AR application. In this regard, information from the visual scene analysis is transferred as input to the WIP in order to provide personalized information about safety to the worker (arrow D in Figure 4).

**Figure 7:**
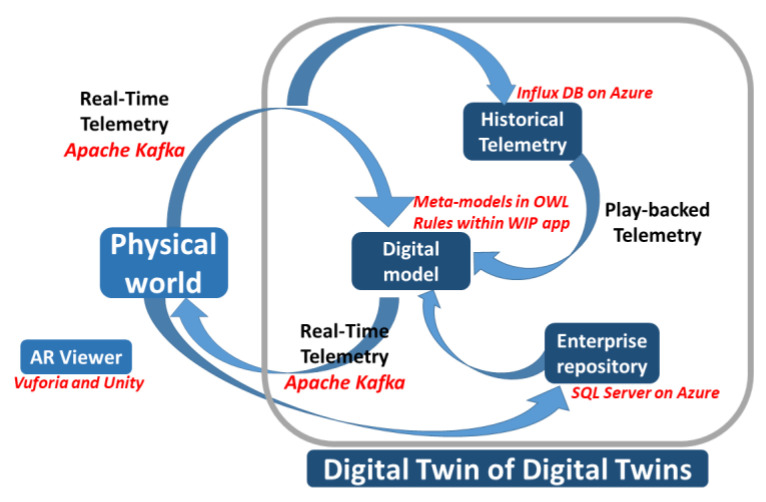
Adopted technological solutions for each logical component of the DT architecture.

It should also be noted that the published information is maintained in a repository to keep track of the evolutions occurring within the whole system, and this information is exploited to customize the experience of the workers. For telemetry, the adopted repository is InfluxDB [53], a commercial time-series database solution meeting the requirements typical of an intensive data scenario [54], while for the enterprise repository, the Sql server is chosen [55]. Moreover, in order to guarantee the horizontal scalability of the overall architecture, InfluxDB is deployed into a cloud-based platform, which is Microsoft Azure [56]. The overall implementation of the analyzed case study is summarized in Figure 7, which illustrates the adopted technological solutions (underlined in red) for each logical component of the DT architecture represented in Figure 1.

### 5.3. Discussion of Results

In order to validate the solution implemented for the case study, two groups of fifteen workers were selected, one group comprising very experienced users and the second comprising users with average experience.

**Figure 8:**
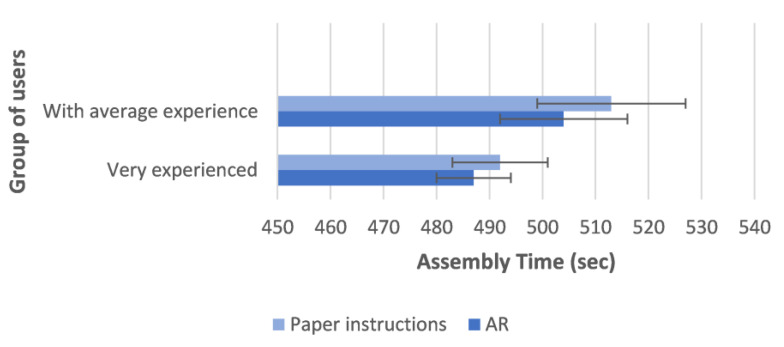
Average assembly time per user group.

**Figure 9:**
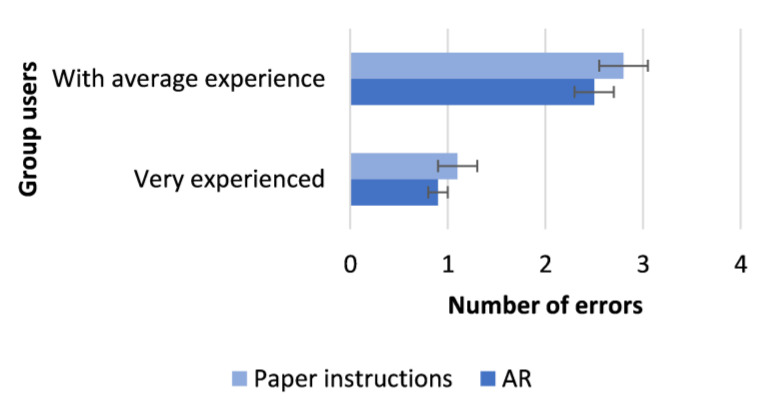
Average assembly errors per user group.

These workers were asked to perform six different assembly activities using both the traditional approach (hard-copy manuals for work instructions) and the AR application. Their performance corresponding to these two approaches was assessed and compared by exploiting the two following quantitative metrics: (1) the overall time taken to complete the task; (2) the number of errors made in the execution of the task (e.g., the use of the wrong component, incomplete assembly, or incorrectly assembled component). Therefore, during the experiment, these two metrics were monitored in both approaches (the AR solution and paper instructions), and the results of the average assembly times and the average of the generated errors are plotted in Figure 8 and Figure 9, respectively, leveraging the histograms. Each plotted histogram also represents the standard deviation. In this regard, it should be noted that the value of the standard deviation was in all cases rather low, and therefore the deviation of the various data from the average was always minimal.

It is apparent from these two figures that the AR solution based on CENTRO and HDT enhanced the quality of the assembly processes since this approach outperformed the traditional approach in terms of each of the two selected metrics. In particular, the achieved results demonstrated that the workers (both the very experienced group and those with average experience) can perform tasks faster and make fewer mistakes if they adopt the proposed solution, which provides personalized AR-based instructions during their assembly activities.

In addition, besides these quantitative results, qualitative results were also obtained through a specific survey administered to the thirty users involved in the experiment, with the aim to elicit their feedback. The survey questionnaire comprises the following three questions:

Q1. With which of the two approaches (AR-based solution or with the paper instructions) do you feel more confident in carrying out the assembly?

Q2. Which of the two approaches supports you best in picking the next component to assemble?

Q3. Which of the two approaches guides you best in assembling a new component with the already assembled part?

The results of the survey confirm the positive results of the quantitative analysis. Specifically, regarding Q1, 74% of the surveyed users felt more confident with the AR solution. Regarding Q2, 80% of the users indicated that the AR application provides clearer support in choosing the next component. Finally, in terms of Q3, 90% of the surveyed users stated that the AR application provides better guidance on how to assemble the new component.

Finally, the software infrastructure’s performance was evaluated in the pilot study. In this regard, the test results of the solution demonstrate the overall system’s scalability, as it could support high throughput for telemetry (up to 500 KB per second) with a high number of connected resources (up to 500, comprising 450 publishers and 50 subscribers, with each publisher using a rate of 1 s).

## 6. Concluding Remarks

In this paper, we explored a strategy to implement CI interactions in the manufacturing field according to the principles of the Industry 5.0 paradigm. The overall aim was to identify practical operational lines to implement a human-centric approach in factories, thus contributing to overcoming the lack of significant human-centered practical cases in the current state of the art.

A major result of this research is Human-CENTRO, a reference architecture for an application that enables a model of CI interaction between humans and machines. Moreover, the validity of the framework was confirmed by assessing the potential of its implementation in a real case study. In detail, Human-CENTRO was exploited to implement a user-centered environment within the factory tailored to the specific needs of workers and able to ensure their well-being and continuous training without losing productivity. In this regard, we described the architectural components adopted in this case study to support the framework.

The Human-CENTRO’s functionalities can be exploited in all the fields where reciprocal situational assistance between machines and humans is required. One of these fields is robotics, where CI can be an enabling pattern to realize mutual situational assistance between machines and robots. In addition, Human-CENTRO can be exploited in fields other than manufacturing (e.g., in medicine to support the interaction between surgeons and machines) since the transfer technology of the framework is easy to apply and can be used with little effort.

### Future Steps

Future studies and developments related to this work will mainly address the following four goals:

i. Human-CENTRO will be further assessed within other experiments set in different industrial fields in order to explore the generalizability of the framework. This evaluation is currently ongoing.
ii. A digital factory belonging to the AI REGIO network will be adapted considering Industry 5.0 principles. In this context, the Human-CENTRO model will also be tested and evaluated.
iii. The PT model will be extended through the in-depth specification of the interactions between internal PT’s macro-components and, in particular, between the digital person model representing the information of the physical worker and the behavioral/cognitive model and computational intelligence.
iv. Data sovereignty mechanisms will be adopted to support the proposed DT model. Such a solution should support the following:
  a. Scenarios of secure data exchange between DTs (or models) internal to the organization;
  b. Scenarios where an organization exposes a DT to external stakeholders within a virtual and trusted data space.

## Figures and Tables

**Table 1:** The definition of each arrow illustrated in Figure 2.

Arrow	Description
1	Indicates an interaction of the CI pattern “Machines Assist Humans”. Examples of this interaction can be realized through an exoskeleton or AR viewer.
2	Indicates an interaction of the CI pattern “Humans Assist Machines”. An example consists of the transfer of the expert domain knowledge in order to train the machine learning model of the DTA.
3	Indicates an interaction of the CI pattern “Humans Assist Machines”. An example consists of the selection and transfer of a subset of the functionalities of cognitive models of the PT to the DT of the system.
4	Represents a physical interaction that is not part of the set of CI interactions. It links the physical worker with the physical system. For example, this interaction can occur when the worker acts on a haptic device, a mechanical device, or even a keyboard.

**Table 2:** Enabling technologies of CI pattern interactions.

	Machine Assists Human	Human Supports Machine
**Physical machine**	- Exoskeleton [40], robot [41] (arrow 1) - AR viewer [42] (arrow 1)	
**Digital machine**		- Transfer knowledge through voice/digital interaction [43] (arrow 2) - Cognitive model [44] (arrow 3)

**Table 3:** The definition of each arrow illustrated in Figure 3.

Arrow	Description
A	Corresponds to the factory telemetry component. It enables the process of synchronization between the physical asset and its DT. Examples: (a) the data corresponding to the updated positions of the assets within the shop floor; (b) assuming the asset is rotating machinery, measurements of machine vibrations, which are reported in terms of displacement, velocity, and acceleration.
B	Represents a new telemetry stream that enables the synchronization between the physical worker and its digital personal twin. Examples: this telemetry stream can include, for example, the data corresponding to the position of the worker on the shopfloor or their posture or also the parameters that measure the stress and fatigue of the worker.
C	Supports the pattern “Machines Assist Humans” indicated by arrow 1 in Figure 2. Allows the information from a physical device to be conveyed to the worker. Example: the real-time information concerning production processes (e.g., the state of the ongoing operations), which is visualized using an augmented reality viewer or a CNC machine display.
D	Enables the interplay between the DTA and the PT and vice versa. Example: when a machine must consider the capabilities and needs of the users and adapt accordingly, the DT of the machine must access the data of each user (contained in their PT) for its processing and elaboration, and thus a bidirectional data pipeline between the PT and the DTA is needed.
E	Supports the pattern “Humans Assist Machines” indicated by arrow 2 in Figure 2. Example: the data model used to transfer expert domain knowledge in order to train the machine learning model of the DTA.
F	Supports the pattern “Humans Assist Machines” indicated by arrow 3 in Figure 2. It represents the transfer of the cognitive models of the PT to the DT of the system. Examples: a cognitive model representing human body experience, which is used in a robotic system to improve its capabilities.
